# Association Between Early Parental Death and Loneliness in Adulthood: A Community-Based Study in Southwest China

**DOI:** 10.3389/fpsyt.2022.784000

**Published:** 2022-03-31

**Authors:** Anjiao Peng, Wanlin Lai, Shixu He, Wanling Li, Tingting Song, Shuming Ji, Xia Zhao, Lei Chen

**Affiliations:** ^1^Department of Neurology, Joint Research Institute of Altitude Health, West China Hospital, Sichuan University, Chengdu, China; ^2^Department of Project Design and Statistics, West China Hospital, Sichuan University, Chengdu, China; ^3^Department of Clinical Research Management, West China Hospital, Sichuan University, Chengdu, China

**Keywords:** early parental death, loneliness, adverse childhood experiences, mental health disorders, mental health problems

## Abstract

Loneliness is a growing public health problem that threatens physical and mental health to a large extent. Compelling evidence has shown that premature parental death is strongly associated with many mental health disorders in adulthood, but whether it increases the risk of loneliness remains unclear. In this large community-based study, we included 32,682 adult participants (20–93 years old) from Southwest China and used the three-item short version of University of California, Los Angeles, Loneliness Scale to identify participants with loneliness. A total of 1,975 participants reported loneliness, which resulted in a loneliness prevalence of 6.0% in Southwest China. Logistic regression was used to evaluate the association between early parental death and loneliness after adjusting for age, gender, education level, marital status, smoking and drinking status, living status, and chronic diseases. We found that early parental death was significantly associated with loneliness [odds ratio (OR) = 1.21, 95% confidence interval (CI), 1.03–1.42]. A Sensitivity analysis excluding those with mental health disorders (796 participants) yielded similar results (OR = 1.26, 95% CI = 1.06–1.49). We also found that being younger, single, divorced, or widowed, and more educated; living alone; and having chronic disorders were associated with loneliness. We conclude that childhood parental death is associated with loneliness in adulthood, suggesting the need for early intervention in affected children to prevent long-term adverse neuropsychiatric effects.

## Introduction

Loneliness is a distressful feeling of social isolation, even if the person is not alone. This is a pervasive mental health condition that has attracted great attention worldwide in recent years (1, 2). Various epidemiological studies have shown that the prevalence of loneliness in middle-aged and older adults ranges from 10.5% up to 78.1% (3–5). Meanwhile, loneliness increases the risk of premature death by 26%, and continues to increase (1). Loneliness has been found to be closely associated with depression (4, 6, 7) and many chronic diseases, such as dementia (8, 9), cardiovascular disease and stroke (10, 11), diabetes, obesity, and respiratory diseases (6, 12). An in-depth understanding of the risk factors for loneliness is essential to better intervene in this condition.

There is compelling evidence suggesting that adverse childhood experiences can generate long-term negative effects on mental health. For example, it increases the risk of alcoholism, problematic drug substance use, depression, anxiety, and other mental health disorders in adulthood (6, 7). Parental death is a traumatic event in childhood that has been associated with depression, suicide, and other chronic illnesses in adulthood (13, 14). However, research on the relationship between early parental death and adulthood loneliness remains limited, and results are inconsistent (15, 16).

To investigate the prevalence of loneliness in Southwest China and to further our understanding of the relationship between childhood parental loss and loneliness in adulthood, we conducted this community-based study of more than 30,000 adults from Sichuan Province, China.

## Materials and Methods

### Participants

Individual-level data were obtained from the Natural Population Cohort Study of West China Hospital of Sichuan University, an ongoing prospective cohort study conducted in communities, with approval from the Ethics Committee of West China Hospital of Sichuan University. Participants older than 20 years from multiple communities (Longquan, Mianzhu, and Pidu) were encouraged to participant in this project. Communities were distributed in rural, urban, and urban–rural borders. The cohort study started in Longquan in 2019 (with the first wave in 2019 and the second in 2020), in Mianzhu in 2020, and in Pidu in 2021. Annual follow-ups were conducted after initiation. This study used data from Longquan (collected in 2020), Mianzhu, and Pidu (collected in 2021), and data analysis was completed in August 2021.

### Loneliness Assessment

Loneliness in adulthood was assessed using the three-item short version of the University of California, Los Angeles (UCLA), Loneliness Scale (17). The scale evaluated the frequency of lack of companionship, exclusion, and isolated from others. Response categories ranged from “never” = 1, “sometimes” = 2, to “too often” = 3. The total score ranges from 3 to 9, with higher scores indicating greater loneliness and a score of 6 or above being classified as lonely (18).

### Parental Death and Covariates

All participants were asked whether they had experienced parental death before the age of 17 years, and the answers were yes and no. The following factors were used as covariates: age, gender, marital status, education level, living status, drinking and smoking status, and chronic diseases. Marital statuses included married, single, divorced, and widowed. Educational levels were categorized as elementary school, middle school, high school, and college (or above). Smoking statuses were categorized as yes and no, similar to drinking statuses. Chronic diseases prediagnosed by physicians, including hypertension, diabetes (including intermediate hyperglycemia and prediabetes), hyperlipidemia, stroke, cancer, heart diseases (including coronary heart disease, myocardial ischemia, bundle branch block, and heart failure), respiratory diseases (including chronic obstructive pulmonary disease, asthma, chronic bronchitis, and emphysema), and mental health disorders (depression and anxiety).

### Depression and Anxiety

Considering that anxiety and depression are largely underdiagnosed and underreported in China (19, 20), we also used validated scales in this study to screen participants with anxiety (or depression). The 9-item Patient Health Questionnaire (PHQ-9) was used to screen participants for depression, and the 7-item Generalized Anxiety Disorder Scale (GAD-7) was used for anxiety screening. The total score of PHQ-9 ranges from 0 to 27, with a score of 10 or more for major depressive disorder (21). The total score of GAD-7 ranges from 0 to 21 points, and participants with a score of 10 or higher were considered to have anxiety disorders (22).

### Statistical Analysis

Continuous variables were presented as mean and standard deviation; categorical variables were described as frequencies and percentages. Student *t*-test was used to analyze continuous variables; a χ^2^ test was used for categorical variable. Logistic regression models were used to evaluate odds ratios (ORs) and 95% confidence intervals (CIs) for the association between early parental death and loneliness. Two models were performed: model 1 adjusted for age and gender and model 2 adjusted for all potential confounders, including age, gender, marital status, education level, living status, smoking and drinking status, and chronic diseases. We adjusted for age and gender only in model 1 to avoid overadjustment.

Considering that loneliness symptoms (lack of companionship, having been left out, and isolated from others) screened with UCLA-3 may also be present in some participants with depression or other mental health disorders, we conducted sensitivity analyses excluding participants with depression and anxiety in addition to primary analyses. In this study, those who had been previously diagnosed by physicians or screened positive for the scale were excluded.

We also performed subgroup analyses to explore the relationships between early parental loss and loneliness in the younger (<60 years) and older (≥60 years) groups. In these subgroup analyses, logistic regression models were adjusted for all confounders. Statistical analyses were performed using SPSS (IBM, version 25) and R 4.0 (R Project for Statistical Computing), and a two-sided *p* < 0.05 was considered statistically significant.

## Results

### Characteristics of the Participants

**FIGURE 1:**
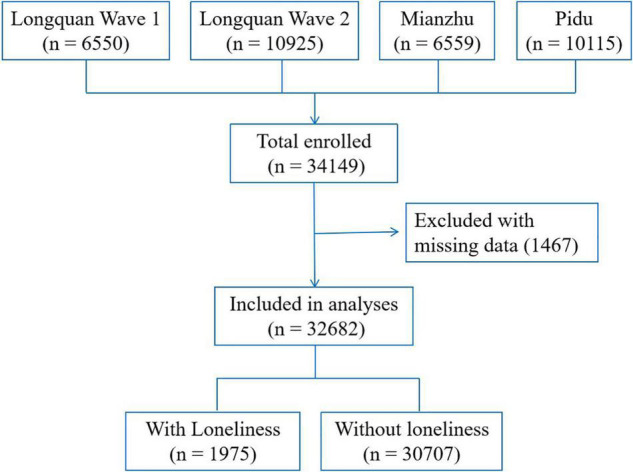
The flowchart of participant inclusion.

The current cohort enrolled 34,149 participants (including 6,550 participants in the first wave of Longquan, 10,925 participants in the second wave of Longquan, 6,559 from Mianzhu, and 10,115 in Pidu). A total of 1,467 (4.3%) were excluded because of missing data. Finally, 32,682 participants (21,685 female and 10,997 male, age range = 20–93 years) were enrolled in the study, of whom 3,410 (10.4%) reported early parental death. The participant inclusion flowchart is shown in Figure 1. Among all included participants, 1,975 [6.0% 95% CI (5.8–6.3%)] felt lonely, and those who felt lonely were younger than those who did not (49.2 ± 13.7 vs. 56.3 ± 11.7 years, *p* < 0.001) (Table 1).

**TABLE 1:** Characteristics of participants with or without loneliness and respective odds.

	No loneliness (*n* = 30,707)	Loneliness (*n* = 1,975)	*p-*value	OR (95% CI), age/sex adjusted	OR (95% CI), full adjustment
Age^a^, mean ± SD, years	56.3 ± 11.7	49.2 ± 13.7	<0.001	–	–
**Age groups, *n* (%)**			<0.001		
≤29 years	609 (2.0)	144 (7.3)		Indicator	Indicator
30–39 years	2,179(7.1)	410 (20.8)		0.80(0.65−0.98)	1.07(0.83−1.38)
40–49 years	4,870(15.9)	429 (21.7)		0.37(0.30−0.46)	0.69(0.53−0.90)
50–59 years	11,624(37.9)	545 (27.6)		0.20(0.16−0.24)	0.39(0.30−0.51)
60–69 years	7,374(24.0)	296 (15.0)		0.17(0.14−0.21)	0.33(0.25−0.44)
≥70 years	4,051(13.2)	151 (7.6)		0.16(0.13−0.20)	0.27(0.19−0.37)
Gender (male), *n* (%)	10,389(33.8)	608 (30.8)	0.005	0.93(0.84−1.02)	0.95(0.84−1.09)
**Educational level, *n* (%)**			<0.001		
Primary	11,343(36.9)	482 (24.4)		Indicator	Indicator
Middle	11,699(38.1)	575 (29.1)		0.95(0.83−1.08)	0.97(0.85−1.11)
High	4,631(15.1)	371 (18.8)		1.37(1.17−1.59)	1.43(1.22−1.67)
College or above	3,034(9.9)	547 (27.7)		2.08(1.76−2.45)	2.02(1.70−2.40)
**Marital status, *n* (%)**			<0.001		
Married	27,630(90.0)	1,615(81.8)		Indicator	Indicator
Single	372 (1.2)	98 (5.0)		1.66(1.26−2.19)	1.13(0.84−1.53)
Divorced	827 (2.7)	124 (6.3)		2.36(1.93−2.88)	1.82(1.46−2.25)
Widowed	1,878(6.1)	138 (7.0)		2.07(1.70−2.52)	1.76(1.43−2.17)
Smoking (yes), *n* (%)	5,090(16.6)	318 (16.1)	0.582	1.01(0.87−1.18)	0.99(0.85−1.16)
Drinking (yes), *n* (%)	7,623(24.8)	528 (26.7)	0.057	1.11(0.99−1.25)	1.06(0.94−1.20)
Live alone, *n* (%)	1,767(5.8)	224 (11.3)	<0.001	2.35(2.02−2.73)	1.84(1.55−2.18)
Early parental death, *n* (%)	3,214(10.5)	196 (9.9)	0.444	1.22(1.04−1.43)	1.21(1.03−1.42)
Hypertension, *n* (%)	5,503(17.9)	250 (12.7)	<0.001	1.03(0.89−1.19)	0.97(0.84−1.13)
Diabetes, *n* (%)^b^	2,396(7.8)	121 (6.1)	0.007	1.09(0.90−1.32)	1.04(0.85−1.27)
Hyperlipidemia, *n* (%)	802 (2.6)	48 (2.4)	0.623	1.18(0.87−1.58)	1.00(0.73−1.36)
Heart diseases, *n* (%)^c^	450 (1.5)	30 (1.5)	0.848	1.47(1.01−2.14)	1.34(0.91−1.98)
Respiratory diseases, *n* (%)^d^	1,098(3.6)	79 (4.0)	0.327	1.37(1.08−1.74)	1.29(1.01−1.65)
Stroke, *n* (%)	564 (1.8)	39 (2.0)	0.659	1.66(1.19−2.32)	1.45(1.02−2.05)
Cancer, *n* (%)	398 (1.3)	17 (0.9)	0.094	0.84(0.51−1.37)	0.79(0.48−1.30)
**Mental health disorders**					8.75(7.43−10.30)
Depression (prediagnosed), *n* (%)	83 (0.3)	32 (1.6)	<0.001	–	–
Depression (screened by PHQ-9), *n* (%)	178 (0.6)	184 (9.3)	<0.001	–	–
Anxiety (prediagnosed), *n* (%)	36 (0.1)	13 (0.7)	<0.001	–	–
Anxiety (screened by GAD-7), *n* (%)	291 (0.9)	172 (8.7)	<0.001	–	–

*CI, confidence interval; GAD-7, 7-item Generalized Anxiety Disorder Scale; OR, odds ratio; PHQ-9, 9-item Patient Health Questionnaire; SD, standard deviation. ^a^Age was included as ranked variables in age-/gender-adjusted model and full adjustment model. ^b^Including intermediate hyperglycemia and prediabetes. ^c^Heart diseases included coronary heart disease, myocardial ischemia, bundle-branch block, and heart failure. ^d^Respiratory diseases included chronic obstructive pulmonary disease, asthma, chronic bronchitis, and emphysema.*

### Association Between Early Parental Death and Loneliness

From the results, it can be seen that in the age- and sex-adjusted model (OR = 1.22, 95% CI = 1.04–1.43) and the fully adjusted model (OR = 1.21, 95% CI = 1.03–1.42) (Table 1), those whose parents died early were more likely to feel lonely. In sensitivity analyses that excluded people with mental health disorders (796 participants, including 477 participants for depression and 512 participants for anxiety), parental death was still found to be associated with loneliness in age- and sex-adjusted model (OR = 1.29, 95% CI = 1.09–1.52) as well as fully adjusted model (OR = 1.26, 95% CI = 1.06–1.49).

Subgroup analyses showed that early parental death was significantly associated with loneliness in younger adults (OR = 1.34, 95% CI = 1.09–1.65), whereas this effect faded in older adults (OR = 1.06, 95% CI = 0.82–1.36).

### Other Factors Associated With Loneliness

In age- and gender-adjusted model, several factors were found to be associated with loneliness. Older participants tended to feel less lonely than younger participants (OR = 0.16, 95% CI = 0.13–0.20 for participants aged ≥70 vs. ≤29 years). People with higher education were more likely to feel lonely (OR = 1.37, 95% CI = 1.17–1.59 for those who attended high school and OR = 2.08, 95% CI = 1.76–2.45 for those who attended a college or above compared with those who attended elementary school or illiterate). Individuals who were single, divorced, or widowed had a higher risk of feeling lonely (OR = 1.66, 95% CI = 1.26–2.19; OR = 2.36, 95% CI = 1.93–2.88; and OR = 2.07, 95% CI = 1.70–2.52, respectively) compared with those who were married. People living alone were more likely to feel lonely (OR = 2.35, 95% CI = 2.02–2.73). Smoking and drinking were not found to be associated with loneliness. For chronic diseases, heart diseases, respiratory diseases, and stroke were significantly associated with a higher risk of loneliness (OR = 1.47, 95% CI = 1.01–2.14; OR = 1.37, 95% CI = 1.08–1.74; and OR = 1.66, 95% CI = 1.19–2.32, respectively). Hypertension, diabetes, hyperlipidemia, and cancer were not found to be associated with loneliness (Table 1).

## Discussion

The study, based on more than 30,000 community participants, demonstrated that the prevalence of loneliness in Southwest China was close to 6.0% and that early parental death was associated with loneliness in adulthood, even accounting for other potential risk factors.

The prevalence of loneliness in our study is relatively lower than most previous studies, such as 10.5% reported in German (3), 21.7% reported in Sweden (4), 43% reported in the United States (23), and 78.1% reported in Anhui, China (5). These results are similar to those for other mental disorders. For example, previous surveys, together with our results, suggest that the prevalence of depression and anxiety in Sichuan is much lower than in other provinces (24, 25) and other countries (26, 27). Taken together, these results may suggest that people in Sichuan are more optimistic than those in other regions, leading to a relatively lower rate of loneliness. What is more, the relatively lower prevalence of loneliness in our study could be partly explained by the timing of this study. Our survey was conducted in 2020 and 2021, nearly one year after the COVID-19 pandemic. People during this time may be more united and value their lives than ever before, which may also lead to lower rates of loneliness than studies from other periods.

The most important finding in our study is that we found that early parental death is associated with loneliness. There is growing evidence suggesting that, in addition to parental death, adverse childhood experiences, such as sexual abuse, physical abuse, and neglect, can significantly impair long-term physical and psychological health and increase the risk of loneliness and mental disorders in adulthood (6, 7, 13, 14, 28, 29). Loneliness has also been found to mediate the association between adverse childhood experiences and mental diseases, such as depression and anxiety (29). *Vice versa*, mental health problems can also increase the risk of loneliness; for example, having depression, anxiety, or other mental disorders can cause people to feel stigmatized and reduce their social interactions with others (28).

Importantly, in this study, the association between parental death and loneliness remained statistically significant after excluding people with mental illness, suggesting that early parental death may be an independent risk factor for adulthood loneliness. Previous researches have shown that parent–infant attachment and healthy early experiences are vital for the development of self-confidence, resilience, social competence, optimism, and trust during one’s growth, whereas negative experiences can affect social support systems in adulthood (29, 30). Thus, people may be more likely to develop certain personality traits that affect their ability to form other relationships (30), resulting in higher rates of loneliness. Interestingly, we found that the effects of early parental death on loneliness were not significant in older adults compared with younger adults, suggesting that the effects of negative childhood experiences on mental health may diminish with age.

Given the profound and long-term health effects of early parental death, those affected need professional support to increase their resilience and reduce the impact of negative impacts on them. In China, all children are required to receive 9 years of compulsory education (i.e., mostly from the age 6 to 15 years). Therefore, schools are where young children stay the most and are the ideal place for intervention. Specialized departments can be set up to provide psychological counseling and support for children whose parents have passed away.

Consistent with previous study, we found that being single, divorced, or widowed; living alone; and having cardiocerebrovascular diseases were associated with loneliness (5, 28, 31). However, previous findings on the association between age and loneliness have been inconsistent across studies (3, 32, 33). Consistent with two previous studies (3, 32), we found that age was negatively correlated with loneliness. As older adults are more satisfied with their friendships and life (34, 35), this may explain why older adults are less likely to feel lonely. We also found an inverse association between education level and loneliness, which is consistent with a recent study conducted in Turkey (36), but contrary to the findings of several previous studies (5, 31). Considering that older and less educated individuals are more likely to be satisfied with their lives (35), increased overall life satisfaction may also be associated with a reduction in their loneliness.

There are some limitations needed to be mentioned in this study. First, recall bias and reporting bias could not be eliminated because of the cross-sectional nature of the study. Second, we did not collect information on the gender and number of parents who died. The lack of this information limited our further analysis. Finally, the results of this study, conducted in Southwest China do not represent the whole picture of the country.

## Conclusion

The findings of this study suggest that parental death in childhood is associated with loneliness in adulthood, suggesting the need for early intervention to prevent long-term detrimental neuropsychiatric effects.

## Data Availability Statement

The raw data supporting the conclusions of this article will be made available by the authors, without undue reservation.

## Ethics Statement

The studies involving human participants were reviewed and approved by the West China Hospital of Sichuan University. The patients/participants provided their written informed consent to participate in this study.

## Author Contributions

AP designed the study, conducted the study, carried out the statistical analysis, and drafted the manuscript. WLi, TS, and XZ collected the information of participants. WLa and SH drafted the manuscript. SJ carried out the statistical analysis. LC designed the study and revised the manuscript. All authors contributed to the article and approved the submitted version.

## Conflict of Interest

The authors declare that the research was conducted in the absence of any commercial or financial relationships that could be construed as a potential conflict of interest.

## Publisher’s Note

All claims expressed in this article are solely those of the authors and do not necessarily represent those of their affiliated organizations, or those of the publisher, the editors and the reviewers. Any product that may be evaluated in this article, or claim that may be made by its manufacturer, is not guaranteed or endorsed by the publisher.
